# Editorial: Microbial solutions for soil health and remediation: from natural diversity to engineered communities

**DOI:** 10.3389/fmicb.2026.1947537

**Published:** 2026-08-14

**Authors:** Rui Zhuo, Xuan Zhang

**Affiliations:** 1 Hunan Province Key Laboratory of Plant Functional Genomics and Developmental Regulation, and Hunan Research Center of the Basic Discipline for Cell Signaling, College of Biology, Hunan University, Changsha, China; 2 State Key Laboratory of Woody Oil Resources Utilization, Hunan Academy of Forestry, Changsha, China

**Keywords:** microbial inoculants, plant-microbe interactions, pollutant bioremediation, soil microbiome, synthetic microbial communities

Soils are living ecosystems that sustain food production, biodiversity, nutrient cycling, and environmental quality. The soil microbiome plays a central role in these processes, yet its enormous diversity and functional complexity remain difficult to fully resolve (Lee et al., 2025). Increasing pressures from intensive agriculture, land degradation, heavy metal and metalloid contamination, pesticide residues, microplastics, and ecosystem disturbance are reshaping soil microbial communities and weakening the ecological functions they support (Chen et al., 2022; Deng et al., 2024). Because soil microorganisms connect plant productivity, ecosystem functioning, environmental quality, and broader One Health outcomes, microbiome-based strategies are increasingly recognized as promising tools for restoring soil health and improving ecosystem resilience (Bahram et al., 2018).

The Research Topic “*Microbial Solutions for Soil Health and Remediation: From Natural Diversity to Engineered Communities*” was established to highlight recent progress in discovering functional microorganisms, understanding microbiome assembly, and developing microbiome-based strategies for restoring soil health and remediating contaminated environments.

A first theme emerging from this Research Topic is the value of natural, degraded, and underexplored environments as reservoirs of functional microbial resources. Fang et al. isolated plant growth-promoting bacteria from Pisha sandstone regions, a fragile and erosion-prone ecosystem in the Yellow River Basin. These strains possessed phosphate solubilization, nitrogen fixation, indole-3-acetic acid production, and siderophore production capacities, and the combined inoculant of F11 and G1 improved soil nutrient availability and promoted the growth of *Medicago sativa* and *Astragalus laxmannii* in nutrient-poor substrates. In a complementary context, Saxena et al. explored tropical evergreen forest soils as a source of potassium-solubilizing bacteria, showing that *Bacillus tequilensis* NPH2 exhibited strong potassium solubilization, while *Priestia aryabhattai* NPH4 enhanced potassium and phosphorus uptake in maize. Together, these studies emphasize that natural microbial diversity, especially from stress-adapted habitats, provides a foundation for developing biofertilizers and restoration inoculants.

Beyond strain discovery, several studies demonstrate how microbial interventions can improve productivity and nutrient cycling in agricultural or cultivation systems. Xie et al. conducted a 3-year, two-site field experiment evaluating a composite microbial fertilizer containing *Bacillus subtilis* and *Trichoderma harzianum*. The microbial fertilizer increased maize yield, enhanced straw decomposition, improved available phosphorus, and reshaped bacterial and fungal community composition, with effects depending on local agroecological context and native microbial assemblages. Zhang et al. further showed that peat-amended casing soil promoted the production of *Oudemansiella raphanipes* by regulating the casing soil microbiome. The 70% peat treatment achieved the highest yield, increased bacterial richness, enriched beneficial taxa such as *Paenisporosarcina*, enhanced chemoheterotrophy and nitrogen fixation functions, and strengthened microbial network robustness. These studies suggest that successful microbiome management depends not only on introduced microorganisms, but also on substrate properties, soil structure, nutrient availability, resident microbial networks, and ecological compatibility. This perspective is consistent with the growing recognition that microbial inoculants may act as modulators of indigenous plant- and soil-associated microbiomes (Kerner et al., 2023).

A second major contribution of this Research Topic lies in advancing our understanding of plant-mediated microbiome assembly and ecosystem restoration. Plant-associated microbiomes are shaped by interactions among host traits, microbial communities, and environmental conditions, thereby influencing nutrient acquisition, stress tolerance, and plant health (Lu et al., 2026). Deng N. et al. investigated rhizosphere fungal communities across seven constructed wetland types with distinct vegetation compositions. Their study revealed that different plant species selectively enriched distinct fungal lineages, and that soil pH, salinity, organic matter, total potassium, and available phosphorus were strongly associated with fungal community composition and diversity. Both deterministic and stochastic processes contributed to community assembly, with dispersal limitation identified as the dominant process. In forest ecosystems, Mo et al. examined rhizosphere fungal communities and soil nitrogen and phosphorus dynamics across a stand-age gradient in *Larix kaempferi* plantations. They found that fungal community structure diversified from young to near-mature forests and became more stable in mature stands, while phosphorus cycling-related functional genes were closely aligned with soil nutrient variation. Together, these studies highlight rhizosphere fungi as sensitive indicators and potential contributors of ecosystem restoration, nutrient cycling, and soil health.

Pollutant transformation and remediation represent another important dimension of this Research Topic. Deng Y. et al. evaluated a synthetic microbiota consortium designed for microplastic degradation in highland barley rhizosphere soil. The consortium enhanced polystyrene microplastic degradation, altered rhizosphere fungal diversity and function, and improved several crop nutritional indicators, illustrating the potential of synthetic microbiota to address emerging agricultural contaminants. An et al. used integrated transcriptomic and metabolomic analyses to reveal the mechanisms of selenite reduction by *Rhodococcus qingshengii* PM1 isolated from a selenium-rich mine. Their results identified coordinated responses involving antioxidant defense, glutathione metabolism, sulfur metabolism, Fe–S cluster biosynthesis, lipid remodeling, and redox pathways, providing molecular insight into microbial metalloid detoxification. Yang et al. reviewed the environmental behavior, degradation processes, and ecotoxicological risks of imidaclothiz, a fourth-generation neonicotinoid insecticide, underscoring the need for mechanistic studies of pesticide transformation products and microbially mediated remediation. These contributions expand microbial remediation research from conventional pollutants toward emerging contaminants, metalloids, and complex pesticide residues.

Taken together, the articles in this Research Topic reflect a broader transition in soil microbiology and environmental remediation: from descriptive surveys of microbial diversity toward function-oriented discovery, experimental validation, multi-omics-enabled mechanistic understanding, and rational microbiome engineering. One strength of this Research Topic is its consistent effort to connect microbial functions with practical challenges in agricultural production and environmental remediation. Nevertheless, the long-term stability and broader applicability of many reported effects still require validation across sites and environmental conditions. Future studies should place greater emphasis on long-term and multi-site field validation, the ecological compatibility between introduced microorganisms and native microbiomes, and the biosafety of synthetic communities applied in open environments. Integrating multi-omics, ecological network analysis, predictive modeling, culture-based microbiology, and field trials will be essential for designing robust microbial solutions that are both mechanistically grounded and practically scalable (Karkaria et al., 2021).

In summary, this Research Topic demonstrates that microbial solutions can support soil health and remediation by mobilizing nutrients, promoting plant growth, regulating rhizosphere and soil-associated microbiomes, restoring ecosystem functions, degrading organic pollutants, and transforming toxic metalloids. We thank all contributing authors, reviewers, and editorial staff for their efforts, and hope that this Research Topic will stimulate further development of predictive, scalable, and ecologically informed microbial technologies for healthy soils and restored ecosystems.

## References

[B1] Bahram M. Hildebrand F. Forslund S. K. Anderson J. L. Soudzilovskaia N. A. Bodegom P. M. . (2018). Structure and function of the global topsoil microbiome. Nature 560, 233–237. doi: 10.1038/s41586-018-0386-6 30069051

[B2] Chen L. Zhang X. Zhang M. Zhu Y. H. Zhuo R. (2022). Removal of heavy-metal pollutants by white rot fungi: mechanisms, achievements, and perspectives. J. Clean. Prod. 354:131681. doi: 10.1016/j.jclepro.2022.131681

[B3] Deng S. Zhang X. Zhu Y. Zhuo R. (2024). Recent advances in phyto-combined remediation of heavy metal pollution in soil. Biotechnol. Adv. 72:108337. doi: 10.1016/j.biotechadv.2024.108337 38460740

[B4] Karkaria B. D. Fedorec A. J. H. Barnes C. P. (2021). Automated design of synthetic microbial communities. Nat. Commun. 12:672. doi: 10.1038/s41467-020-20756-2 33510148 PMC7844305

[B5] Kerner P. Struhs E. Mirkouei A. Aho K. Lohse K. A. Dungan R. S. . (2023). Microbial responses to biochar soil amendment and influential factors: a three-level meta-analysis. Environ. Sci. Technol. 57, 19838–19848. doi: 10.1021/acs.est.3c04201 37943180 PMC10702529

[B6] Lee K. K. Liu S. Q. Crocker K. Wang J. Huggins D. R. Tikhonov M. . (2025). Functional regimes define soil microbiome response to environmental change. Nature 644:1028. doi: 10.1038/s41586-025-09264-9 40670792 PMC12390847

[B7] Lu Q. Wang K. Gu S. Ma J. Cui D. Chi Z. . (2026). Siderophore-producing Bacillus and free-living nematodes are associated with soil suppressiveness to banana root-knot nematodes. Nat. Commun. 17:2688. doi: 10.1038/s41467-026-69647-y 41680172 PMC13009492

